# Calcium Signaling in Pancreatic Immune Cells *In situ*

**DOI:** 10.1093/function/zqaa026

**Published:** 2020-10-13

**Authors:** Oleksiy Gryshchenko, Julia V Gerasimenko, Ole H Petersen, Oleg V Gerasimenko

**Affiliations:** 1 Cardiff School of Biosciences, Cardiff University, Cardiff CF10 3AX, UK; 2 Bogomoletz Institute of Physiology, Kyiv 01024, Ukraine

**Keywords:** calcium signaling, exocrine pancreas, pancreatic lobules, acute pancreatitis, pancreatic immune cells, pancreatic macrophages, pancreatic stellate cells, pancreatic acinar cells, P2Y receptors, ATP

## Abstract

Immune cells were identified in intact live mouse pancreatic lobules and their Ca^2+^ signals, evoked by various agents, characterized and compared with the simultaneously recorded Ca^2+^ signals in neighboring acinar and stellate cells. Immunochemistry in the live lobules indicated that the pancreatic immune cells most likely are macrophages. In the normal pancreas the density of these cells is very low, but induction of acute pancreatitis (AP), by a combination of ethanol and fatty acids, markedly increased the number of the immune cells. The principal agent eliciting Ca^2+^ signals in the pancreatic immune cells was ATP, but these cells also frequently produced Ca^2+^ signals in response to acetylcholine and to high concentrations of bradykinin. Pharmacological studies, using specific purinergic agonists and antagonists, indicated that the ATP-elicited Ca^2+^ signals were mediated by both P2Y1 and P2Y13 receptors. The pancreatic immune cells were not electrically excitable and the Ca^2+^ signals generated by ATP were primarily due to release of Ca^2+^ from internal stores followed by store-operated Ca^2+^ entry through Ca^2+^ release-activated Ca^2+^ channels. The ATP-induced intracellular Ca^2+^ liberation was dependent on both IP_3_ generation and IP_3_ receptors. We propose that the ATP-elicited Ca^2+^ signal generation in the pancreatic immune cells is likely to play an important role in the severe inflammatory response to the primary injury of the acinar cells that occurs in AP.

## Graphical Abstract

**Graphical Abstract:**
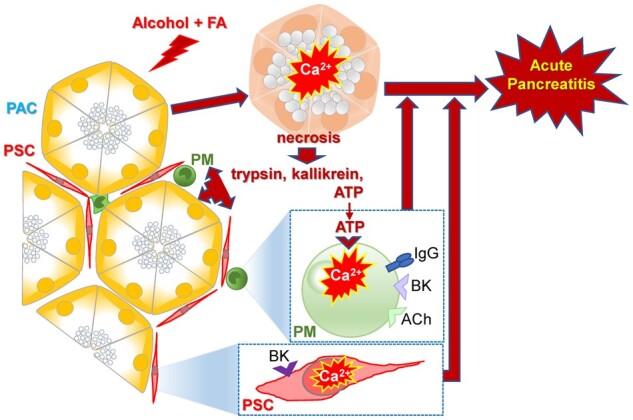

## Introduction

Cytosolic Ca^2+^ signals in the acinar cells of the exocrine pancreas, evoked by acetylcholine (ACh) or cholecystokinin (CCK), control the physiologically important secretion of digestive enzymes and fluid.^1^ The mechanisms responsible for the primary intracellular Ca^2+^ release and the subsequent Ca^2+^ release-activated Ca^2+^ (CRAC) entry of Ca^2+^ are well established.^1–4^ Although the vast majority of Ca^2+^ signaling studies have been conducted on acutely isolated mouse acinar cells or small acinar cell clusters, the general validity of the results obtained has been confirmed by studies in more intact preparations as well as in experiments on human acinar cells.^1^^,^^5^

Whereas local repetitive Ca^2+^ rises regulate physiological secretion, global and sustained elevations of the cytosolic Ca^2+^ concentration ([Ca^2+^]_i_) initiate the disease acute pancreatitis (AP).^6^ Such excessive Ca^2+^ signals can be elicited by a combination of ethanol and long-chain fatty acids, bile acids, or be drug-induced, for example by Asparaginase.^7^ The sustained global elevation of [Ca^2+^]_i_ is generally maintained by open CRAC channels,^2^^,^^6^ but can also occur via pressure-induced Piezo1 activation of TRPV4 channels.^8^

Given that the acinar cells constitute the bulk of the exocrine pancreatic tissue and that these cells synthesize and secrete the digestive enzymes in response to food intake and are responsible for the initiation of AP, it is not surprising that Ca^2+^ signaling studies in the exocrine pancreas have largely been confined to these cells. However, it has recently become clear that other cell types in the exocrine pancreas also play a role, particularly in the pathophysiology of AP.^4^^,^^9–12^

Employing isolated lobules of the exocrine pancreas, in which the normal microscopic structure of the acinar environment is preserved, it has been possible to record simultaneously cytosolic Ca^2+^ signals in several different cell types, including stellate cells and intrinsic nerves.^10^^,^^11^ Whereas the role of the Ca^2+^ signals evoked by ACh and CCK in the acinar cells is well understood, the physiological importance of the Ca^2+^ signals evoked by bradykinin (BK) in the stellate cells^10^ is unclear. There is, however, evidence indicating that BK-elicited Ca^2+^ signals in the stellate cells can magnify the damage to the acinar cells caused by various agents inducing AP, including the combination of alcohol and fatty acids as well as bile acids.^9–11^ At least part of the damaging effect of the BK-elicited Ca^2+^ signals in the stellate cells would appear to be mediated by NO generation, due to Ca^2+^-activation of NO synthase in these cells.^12^

In our recent work on Ca^2+^ signaling in the peri-acinar environment,^11^ we identified, in addition to stellate cells and nerve cells, an unknown cell type, which we called X-cells. These cells displayed prominent Ca^2+^ signals in response to stimulation with ATP and have been the focus of the present study. We now show evidence indicating that these X-cells are pancreatic immune cells, most likely macrophages.

Previously, macrophages of the exocrine pancreas have been studied in culture or fixed tissue, primarily under pathological conditions such as ductal ligation, carbon tetrachloride-induced pancreatitis, and experimental pancreatic cancer.^13^ During pancreatic injury, macrophages infiltrate the tissue leading to inflammation, tissue destruction, and high rates of morbidity and mortality.^14^

There have been limited studies in normal pancreatic tissue that possesses different types of tissue-resident myeloid cells. They could be identified by immunohistochemistry using specific surface proteins including CD11b and F4/80 that are highly expressed specifically in macrophages.^15–17^ It was, however, difficult to identify more precisely myeloid cell subpopulations in the exocrine tissue due to cross-reactivity of conventional dendritic cells and macrophages to the same tissue antigens.^17^

Here we now characterize the Ca^2+^ signaling properties of macrophages *in situ,* using freshly isolated lobules of exocrine pancreas. We demonstrate vigorous Ca^2+^ signal generation induced by ATP as well as some other agents. As ATP will be released from damaged acinar cells in the very early stage of AP, such Ca^2+^ signals could play an important role in the generation of the inflammatory response, which is the major cause of the destruction of the pancreas and surrounding tissues in AP.^18^

## Methods

### Ethical Approval

All animal studies were ethically reviewed and conducted according to the UK Animals (Scientific Procedures) Act, 1986. All animal procedures and experimental protocols were performed under a Project Licence granted by the UK Home Office and approved by the Animal Care and Ethics Committees at Cardiff School of Biosciences, Cardiff University. Animals were maintained in plastic cages supplied with fresh corn cob bedding, tap water, and commercial pelleted diet.

### Induction and Evaluation of Experimental AP

The alcohol-induced experimental model of AP was induced in C57BL6/J mice that received two intraperitoneal (IP) injections of PBS followed by IP injections of a mixture of ethanol (1.35 g/kg) and palmitoleic acid (150 mg/kg) at 1 h intervals as previously described.^11^ We refer to this AP model as FAEE-AP, since fatty acids and ethanol can react together inside cells to produce fatty acid ethyl esters (FAEEs). Control mice received IP injections of the PBS solution alone. Mice were humanely killed by cervical dislocation (Schedule 1) 48 or 72 h after the last injection. For histological assessment pancreatic tissue was fixed in 4% formaldehyde and embedded in paraffin. Fixed slices (4 μm thickness) stained with hematoxylin and eosin, ≥10 random fields of view (magnification: ×200), were evaluated by two blinded independent investigators grading (scale, 0–3; means ± SEM; *n* = 3 mice per group) edema, inflammatory cell infiltration, and necrosis as previously described.^19^

### Lobule Preparation

Pancreatic lobules were freshly isolated from the pancreas of 5- to 7-week-old male C57BL6/J mice^10^ or from mice in which FAEE-AP had been induced as described above. The pancreas was rapidly removed, injected with standard Na^+^-Hepes-based solution containing collagenase and incubated for 5–6 min at 37°C. The standard solution was composed of (in mM): NaCl, 140; KCl, 4.8; Hepes, 10; MgCl_2_, 1; glucose, 10; CaCl_2_, 1 (unless stated otherwise), pH 7.3 (NaOH). The standard solution in experiments for investigation of the effects of membrane depolarisation was modified to contain 100 mM KCl and 44.8 mM NaCl. All experiments were carried out with pancreatic lobules attached to the coverslip of a perfusion chamber at room temperature (∼23°C).

### Ca^2+^ Measurements

Pancreatic lobules were loaded with 5 μM Fluo-4 acetoxymethyl (AM) ester for 20 min at room temperature. The lobules were transferred into a flow chamber and perfused with the standard solution alone or containing different chemicals as described in the experimental protocols of the result section. Cells were visualized using a Leica SP5 MPII two-photon confocal microscope, with an x63 1.2NA objective lens. The Fluo-4 excitation wavelength was 488 nm and emission was collected at 500–560 nm with resolution of 256x256 pixels and speed of 0.7 frames/s. Images were analyzed using Leica Confocal Software (Leica, Mannheim, Germany). Fluorescence signals were plotted as normalized F/F_0_. Control Immunoglobulin G (IgG) was used in concentrations of 0.1–0.25 mg/mL. ANOVA or Student’s *t*-test was performed for statistical analysis.

### Immunostaining in *Ex vivo* Lobules

Immunostaining of live pancreatic lobules was performed as previously described.^10^ Mouse F4/80 and mouse CD11b/Integrin alpha M Alexa Fluor^®^ 647-conjugated monoclonal rat antibodies were used to label specific surface proteins of immune cells, usually at the end of Ca^2+^ measurement experiments, unless otherwise stated. After blocking with 1% BSA and 10% goat serum containing PBS, the isolated pancreatic lobules were incubated for 1 h at room temperature with the selected antibody. Antibody staining was visualized by exciting Alexa Fluor^®^ 647 with 633 nm laser at 10% power and emitted light was collected at 640–700 nm. Hoechst 33342 was used to determine the position of nuclei using excitation 405 nm and collecting light at 420–480 nm. Conjugated antibody fluorescence was also overlaid with Fluo-4 staining as described in the Ca^2+^ measurements section. Lobules were attached to the glass coverslips covered with poly-l-lysin.

### Reagents

BK, S-BK, WIN64338, MeSADP, MRS 2365, MRS 2179, MRS 2211, ADP, SQ 22536 were purchased from Tocris Biosciences (Bristol, UK). GSK7975A was a gift from GlaxoSmithKline (Stevenage, UK). Fluo-4 AM and Hoechst 33342 were purchased from Invitrogen (Life Technologies, Carlsbad, CA, USA). Mouse F4/80 monoclonal rat Antibody (CI-A3-1) [Alexa Fluor^®^ 647] and mouse CD11b/Integrin alpha M Alexa Fluor^®^ 647-conjugated monoclonal rat antibodies were obtained from Novus Biologicals Europe and R&D Systems Bio-techne, respectively. Other chemicals were purchased from Sigma or Calbiochem (Merck, UK).

## Results

### X-cells Identified as Pancreatic Immune Cells

In our previous study,^11^ the unidentified X-cells displayed Ca^2+^ signals that were distinct from those observed in the well-known cells of the pancreatic lobules. One possibility, which we have now investigated, is that the X-cells might be immune cells, as such cells have occasionally been found in pancreatic tissue.^13^^,^^17^

**Figure 1.**
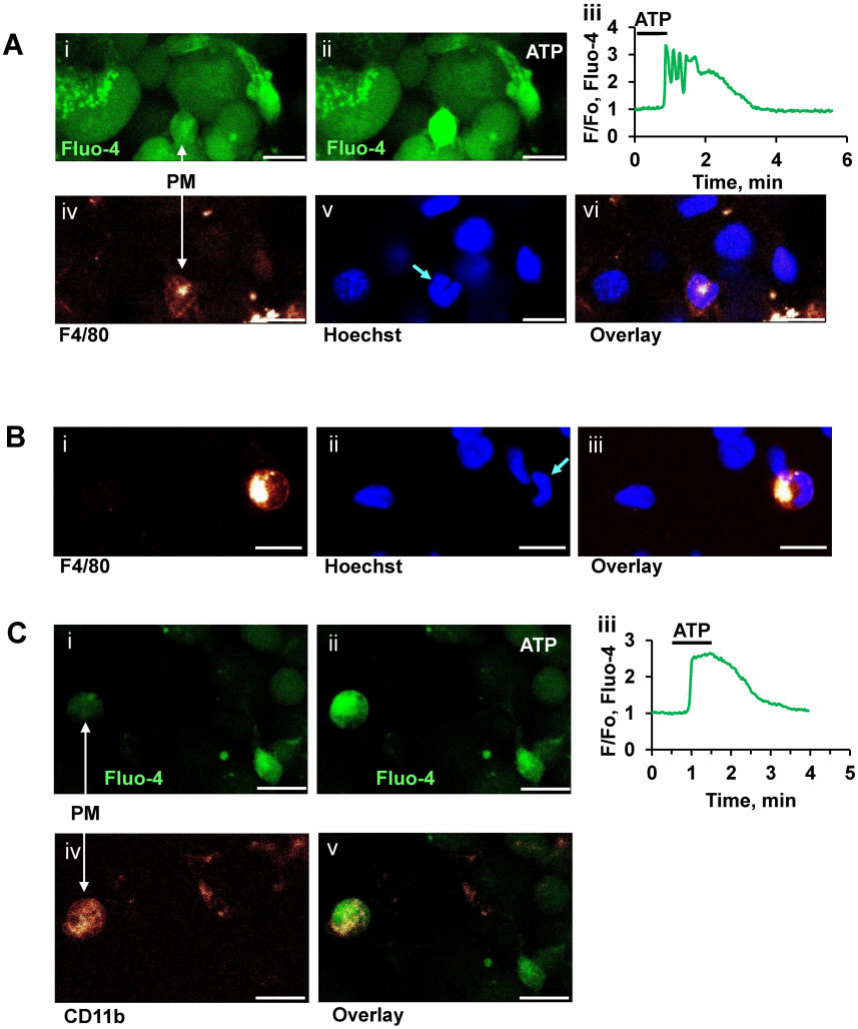
Immunostaining after Recording ATP-elicited Ca^2+^ Signals in pancreatic macrophages (PMs). **(A).** Representative images of pancreatic lobule loaded with Fluo-4AM before **(Ai)** and after ATP (10 µM) application **(Aii),** the arrow indicates the position of the (*n* = 8). A corresponding Fluo 4 trace from a PM is shown in **Aiii**. Corresponding immunostaining of this lobule with antibodies F4/80 Alexa Fluo 647 is shown below (**Aiv)**. Hoechst 33342 staining of the same area is shown in **Av**. Arrow points to ear-like shape of PM nucleus. Overlay of antibody and Hoechst 33342 staining is shown in **Avi**. Scale bar is 10µm. **(B).** Immunostaining of another area in a pancreatic lobule with monoclonal F4/80 antibodies labeled with Alexa Fluor 647 **(Bi)**. Staining of nuclei in the same lobule with Hoechst 33342 **(Bii)**. Overlay of B**i** with **Bii** is shown in **Biii**. Scale bar is 10µm. **(C).** Representative images of a pancreatic lobule loaded with Fluo-4AM before **(Ci)** and after ATP (10 µM) application **(Cii)**, the arrow indicates the position of the PM. Corresponding Fluo 4 trace is shown in **Ciii.** Immunostaining of the same area with monoclonal CD11b antibody conjugated with Alexa Fluor 647 (*n* = 8) is shown in **Civ**. Overlay of **Cii** and **Civ** is shown in **Cv**. Scale bar is 10µm.

**Figure 2.**
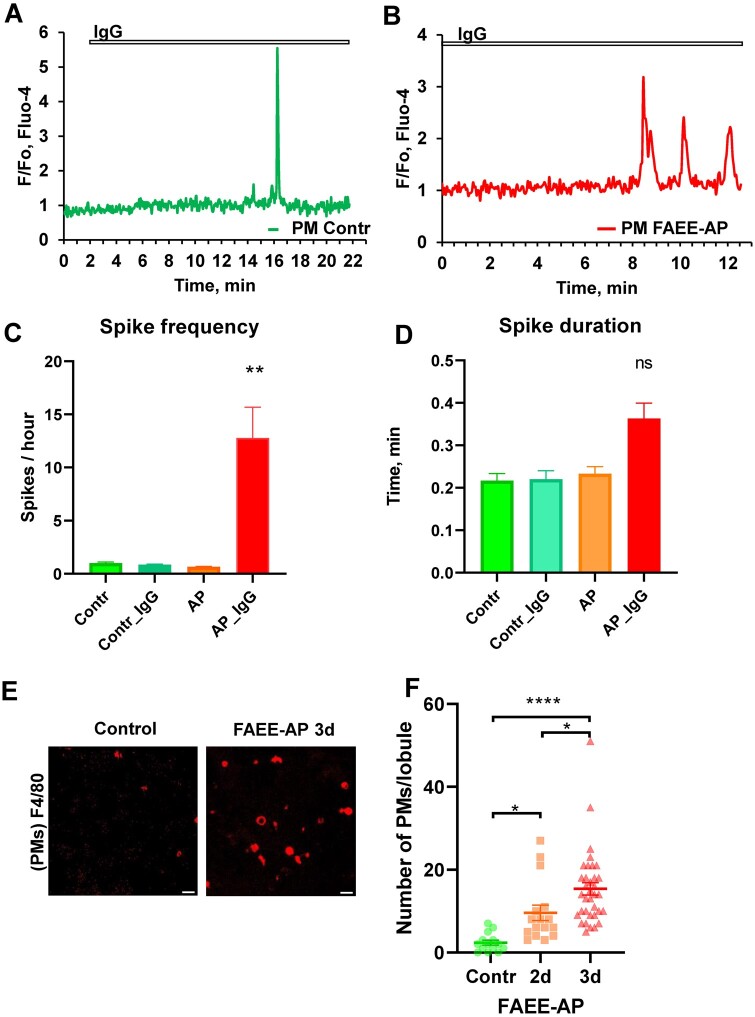
IgG-elicited Ca^2+^ Spikes in PMs**. (A).** Single short Ca^2+^ spike occurring after application of IgG (0.1–0.25 mg/mL) in a PM from a control pancreatic lobule. This was an infrequent observation (5 out of 29 cells tested) and is most likely not an IgG-elicited Ca^2+^ signal as such single spikes have been also observed in 3 out of 15 cells in the absence of IgG stimulation. **(B)**. Representative trace of IgG (0.1–0.25 mg/mL)-induced Ca^2+^ signals in PMs in pancreatic lobules isolated from mice with AP (FAEE-AP model—48 h). Such oscillations were observed in 9 out of 31 cells. Single short spikes have been observed in 4 out of 31 cells. No oscillations were observed in the absence of stimulation with IgG (*n* = 14), while single short spikes have been observed in 2 out of 14 cells. **(C).** Average Ca^2+^ spike frequencies in PMs displaying Ca^2+^ signals under the conditions indicated. The frequencies in control PMs, both stimulated with IgG (blue bar) and unstimulated (green), as well as in unstimulated PMs from the FAEE-AP model (48 h, orange bar) were much lower than in PMs from the FAEE-AP model stimulated with IgG (red bar, *P* < 0.007). **(D)**. Average Ca^2+^ spike duration in PMs displaying Ca^2+^ signals under the conditions indicated. Although the average spike duration was longer in the PMs from the FAEE-AP mice stimulated with IgG than under the other conditions, the difference was not statistically different (*P* > 0.2). **(E).** Representative images of immunostaining of PMs in lobules using antibodies F4/80 conjugated with Alexa Fluor 647. Lobules were isolated from control and FAEE-AP 3-day mice (72 h *in vivo* FAEE-AP model). Scale bar is 20µm. **(F).** Comparison of the average density of PMs in lobules from control and FAEE-AP 2-day and 3-day mice (48 h and 72 h *in vivo* FAEE-AP model, respectively). Control, 2.36 ± 0.6 SEM, *n* = 14; FAEE-AP 2 day, 9.56 ± 1.86 SEM, *P < 0.033, *n* = 16; FAEE-AP 3 days, 15.37 ± 1.51 SEM, *P < 0.038 as compared to FAEE-AP 2-day, *n* = 35. The difference between control and FAEE-AP 3-day was very highly significant (****P < 0.0001).

To test the hypothesis that the X-cells previously described^11^ are immune cells, we employed immunostaining with different antibodies against surface proteins of immune cells labeled with the fluorescent indicator Alexa Fluor 647 (Figures 1A–C and 2D) to allow post staining at the end of functional experiments (Figure1A(iii) and Figure1C(iii)). The fluorescently labeled antibodies F4/80 as well as CD11b have been commonly used to label macrophages.^13^^,^^20^ Staining confirmed the identity of X-cells as pancreatic macrophages (PMs) in the pancreas (Figures 1A–C and 2E). In addition, the ear-like shape of nuclei, typical for macrophages, monocytes, eosinophils, and dendritic cells^21–24^ was very different from the classical round shape of nuclei observed in other cells of the pancreas when stained with Hoechst 33342 (Figure 1A, B).

The control antibodies IgG are known to induce Ca^2+^ spikes in activated immune cells.^25^ We applied IgG to control pancreatic lobules, but did not detect any oscillations. Instead we observed occasionally single Ca^2+^ spikes (Figure 2A), but their appearance was independent of the presence of IgG. We then tested the effect of IgG on PMs in our AP model (FAEE-AP). In lobules isolated from FAEE-AP pancreas (48 h), we detected IgG-induced Ca^2+^ oscillations, after a substantial delay, in about 30% of PMs (Figure 2B). Without IgG stimulation, no oscillations were observed in PMs from FAEE-AP pancreas. These data indicate that PMs are largely quiescent in the pancreatic tissue of control mice, only displaying the occasional spontaneous Ca^2+^ spike. However, after AP induction, PMs become activated^20^ and now close to one-third of the cells respond to IgG stimulation with repetitive Ca^2+^ spiking (oscillations) (Figure 2B, C). The duration of the spikes in the FAAE-AP pancreas appeared to be slightly longer than in the control situation, but the difference was not significant (Figure 2D). The amplitudes of the Ca^2+^ spikes in the FAEE-AP pancreas were not significantly different from those observed in the control pancreas (*P* > 0.06).

Immunostaining of PMs with the fluorescently labeled antibodies F4/80 against surface IC proteins (Figure 1A–C) was used to calculate the relative density of PMs (Figure 2E, F). Whereas the density of pancreatic stellate cells (PSCs) was not different in tissues isolated from control and FAEE-AP (72 h) mice (*n* = 14 and *n* = 31, *P* > 0.54), the density of PMs increased significantly in FAAE-AP mice after 48 h (*P* < 0.033) and was highly significant after 72 h (*P* < 0.0001; Figure 2E, F).

### ATP-elicited Ca^2+^ Signals in PMs

**Figure 3.**
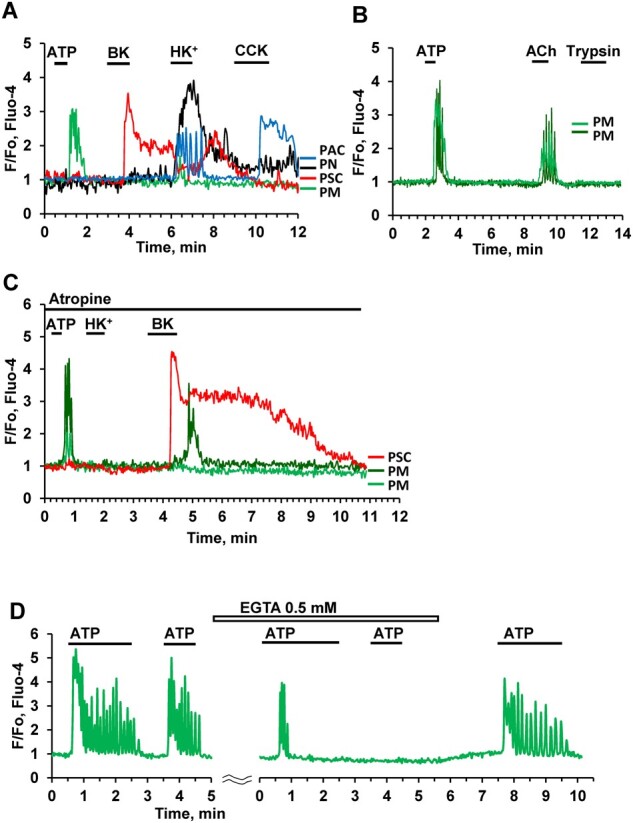
Effects of Stimulation with ATP, Ach, and High K^+^ Concentration. **(A).** Representative traces (normalized [Ca^2+^]_i_ traces, F/F_0_) of simultaneous recordings of [Ca^2+^]_i_ changes in PM (green trace), PSC (red), pancreatic neuron (PN, black), and PAC (blue) in the same lobule. ATP (10 µM) only evoked a Ca^2+^ signal in the PM, whereas a low concentration of BK (20 nM) only elicited a Ca^2+^ signal in the PSC. CCK (at a high concentration, 100 nM) evoked a response in the PAC, but not in the other cells. Exposure to a high K^+^ concentration (100 mM, HK^+^) resulted in an increase in [Ca^2+^]_i_ in all the cells except the PSC. HK^+^ increased [Ca^2+^]_i_ in 31 out of 98 PMs. In the PSC, the HK^+^-induced depolarisation markedly reduced the BK-induced elevated [Ca^2+^]_i_ plateau. This is a consequence of the severely reduced driving force for store-operated Ca^2+^ entry, due to the diminished membrane potential. **(B).** ACh (100 nM) evokes Ca^2+^ signals in two PMs (*n* = 23 out of 33 tested cells) following ATP-elicited Ca^2+^ signals, whereas trypsin had no effect (although trypsin did occasionally induce responses in PMs (3 out of 25 cells). **(C)**. In the presence of atropine (1 µM) HK^+^ (*n* = 20) failed to increase [Ca^2+^]_i_ in two PMs, whereas the effects of bradykinin on both PMs (*n* = 5) and a PSC were retained**. (D).** Addition of 0.5 mM EGTA, in the absence of extracellular Ca^2+^, first reduced and then abolished the response to ATP (10 µM) in a PM (*n* = 5). The ATP response was recovered after removal of EGTA and restoration of the external [Ca^2+^] to 1 mM.

We have previously reported that X-cells, now identified as PMs, are highly responsive to stimulation with micromolar concentrations of ATP.^11^Figure 3A–D demonstrate that PMs (green traces) are capable of generating substantial Ca^2+^ signals in response to ATP (10 μM) (Figure 3A–D).

We tested the possibility that the PMs might be electrically excitable. In these experiments, the cells in the lobule were depolarized by exposure to a solution with a high (100 mM) K^+^ concentration (HK^+^) as previously described.^11^ This evoked short-lasting Ca^2+^ signals in about 30% of the PMs (Figure 3A), but this could be an indirect effect due to release of ACh from depolarized intrinsic nerves (dark blue trace in Figure 3A). In the presence of atropine (1µM) HK^+^ failed to evoke any elevation of [Ca^2+^]_i_ in the PMs, whereas the Ca^2+^ signals evoked by ATP and BK were preserved (Figure 3C). ACh induced Ca^2+^ signals in a majority of PMs (∼70%) (Figure 3B), whereas CCK (Figure 3A) had no effect. Bradykinin elicited Ca^2+^ signals in PMs (in ∼40% of the cases), which were delayed compared to the responses in the neighboring PSCs (Figure 3C).

Depletion of intracellular stores by prolonged stimulation with 10 μM ATP, in the absence of external Ca^2+^ (and in the presence of ethylene glycol-bis(β-aminoethyl ether)-N,N,N′,N′-tetraacetic acid (EGTA) (0.5 mM)), lead to the cessation of Ca^2+^ signaling (Figure 3D). Ca^2+^ oscillations resumed after reintroduction of 1 mM Ca^2+^ to the bath solution and removal of EGTA (Figure 3D).

**Figure 4.**
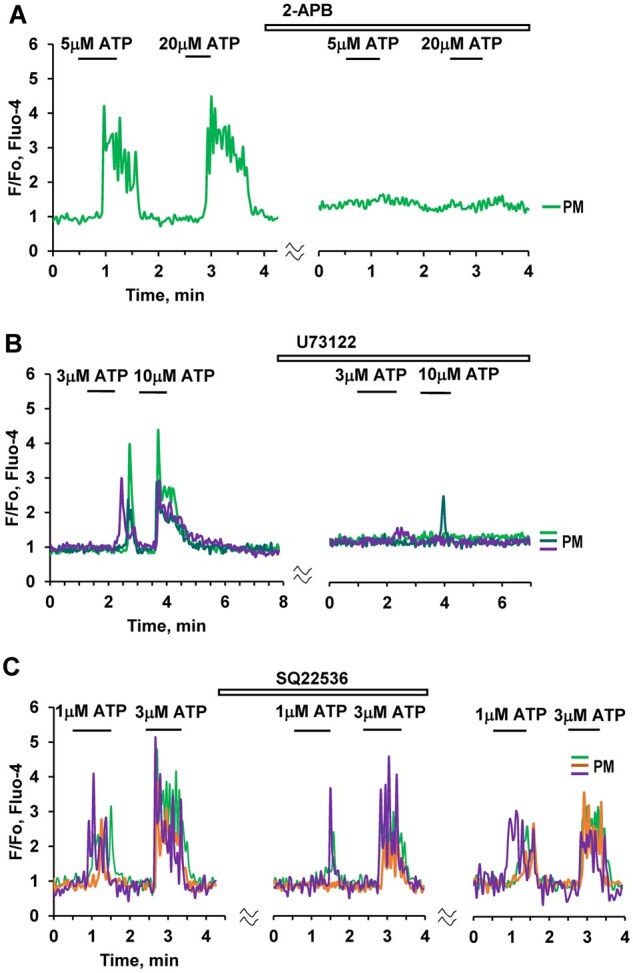
ATP-induced Ca^2+^ Signaling in PMs Depends on IP_3_. (**A**). Ca^2+^ signals induced by ATP at different concentrations (5 µM, 20 µM) are abolished by 100 µM 2-APB (*n* = 5). **(B)**. ATP-elicited Ca^2+^ signals are markedly reduced or abolished by the phospholipase C inhibitor U 73122 (10 μM) (abolished in 36 out of 45 cells)**. (C).** Application of SQ 22536 (200 μM), an inhibitor of adenylyl cyclase, resulted in partial inhibition of Ca^2+^ signals elicited by a low (1 µM) ATP concentration (area under the curve 30–120 s, *P* < 0.02, *n* = 18). Complete inhibition of the response was observed in three cells (brown trace). There was no significant reduction of Ca^2+^ signals evoked by higher concentrations of ATP (3 µM (*n* = 18) or 10 µM ATP (*n* = 12)).

To investigate the potential involvement of inositol trisphosphate (IP_3_) receptors in ATP-elicited Ca^2+^ signaling in PMs, we treated pancreatic lobules with either 100 μM 2-APB (Figure 4A) or the phospholipase C inhibitor U 73122 (10 μM) (Figure 4B). In both cases this resulted in almost complete inhibition of ATP-elicited Ca^2+^ signal generation (Figure 4). In contrast, application of an inhibitor of adenylyl cyclase, SQ 22536 (200 μM), only had a partial but nevertheless significant inhibitory effect on Ca^2+^ signals evoked by 1 μM ATP, but had no effect on the responses to higher doses of ATP, i.e. 3 and 10 µM (Figure 4C).

**Figure 5.**
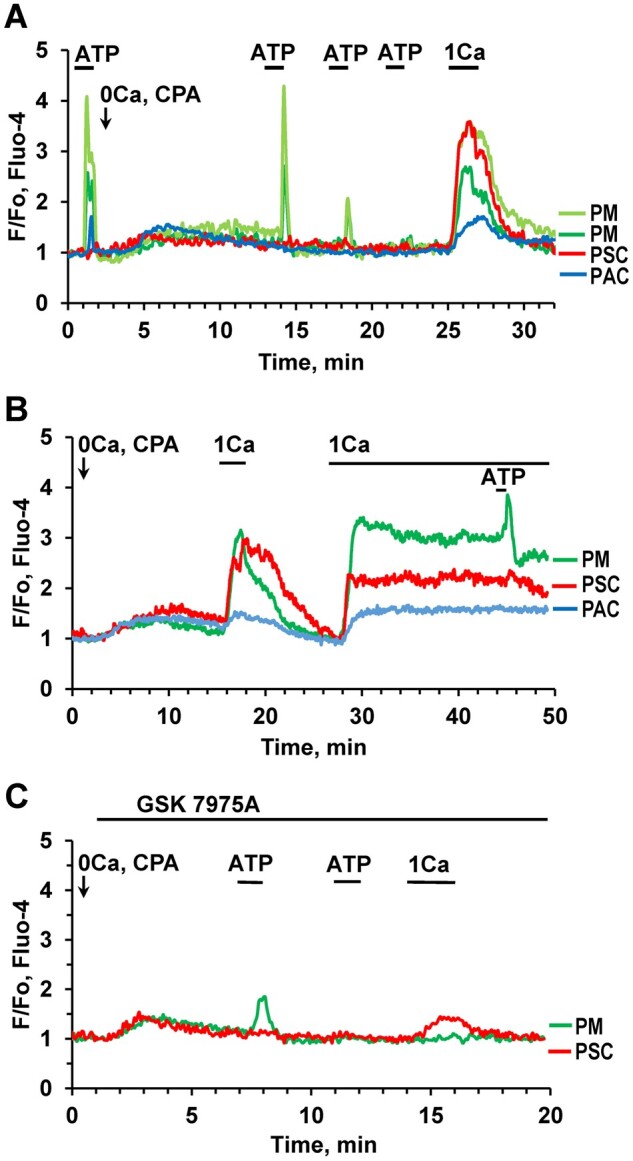
Ca^2+^ Re-entry in PMs after Store Depletion. **(A).** ATP (10 µM)-elicited Ca^2+^ signals in PMs (light green and dark green traces) are gradually lost following exposure to the SERCA inhibitor CPA (20 µM) in the absence of external Ca^2+^. [Ca^2+^]_i_ traces from a PAC (blue) as well as from an ATP-insensitive PCS (red) from the same pancreatic lobule are included for comparison. A short period of external Ca^2+^ (1 mM) readmission resulted in a transient [Ca^2+^]_i_ increase in all four cells (*n* = 6). **(B).** Transient or sustained [Ca^2+^]_i_ rises following short-lasting or permanent readmission of external Ca^2+^ (1 mM) after intracellular Ca^2+^ store depletion by CPA in PM (green trace), PSC (red trace) and PAC (blue trace). ATP (1 μM) elicited a Ca^2+^ signal in the PM on the top of the elevated [Ca^2+^]_i_ plateau following the maintained Ca^2+^ readmission (*n* = 7). **(C).** The CRAC channel blocker GSK7975A (20 μM) abolished store-operated Ca^2+^ re-entry in a PM following Ca^2+^ readmission (*n* = 7).

As mentioned above (Figure 3D), the ATP-elicited Ca^2+^ signals in PMs depend predominantly on Ca^2+^ release from internal stores that can be depleted by prolonged stimulation with ATP in the absence of external Ca^2+^. To study the replenishment of internal stores we have used a standard cyclopiazonic acid (CPA) protocol for Ca^2+^ store depletion, while observing simultaneously four different cells (two PMs, pancreatic stellate cells (PSCs), and Pancreatic acinar cells (PACs); Figure 5A). Application of ATP at the beginning of the experiment, before CPA addition, induced typical Ca^2+^ signals in both PMs, a smaller response in the PACs and no response in the PSCs (Figure 5A). After addition of CPA and removal of external Ca^2+^, there was no response to ATP in the PAC and the ATP-elicited Ca^2+^ signals in the PMs were gradually reduced and finally almost disappeared (Figure 5A). Readmission of external Ca^2+^ (1 mM) induced rapid Ca^2+^ elevations in all cells (Figure 5A) most likely due to store-operated Ca^2+^ entry. In a similar type of experiment (Figure 5B), elevations of [Ca^2+^]_i_ in three different cells (PMs, PACs, and PSCs) were observed after both short and continuous external readmissions of 1 mM CaCl_2_. After readmission of external Ca^2+^ there was also recovery of the ATP-induced response in the PM (Figure 5B). The most likely explanation for the observed rise in [Ca^2+^]_i_ immediately upon external Ca^2+^ readmission is that CRAC channels are open as a result of the intracellular Ca^2+^ store depletion following blockade of the SERCA pumps by CPA.^2^ Antigen stimulation of immune cells is known to trigger Ca^2+^ entry through CRAC channels, promoting the immune response to pathogens.^26^ GSK 7975A (10 μM), a well-known CRAC channel blocker,^2^ abolished Ca^2+^ readmission-induced Ca^2+^ entry in the PM (Figure 5C, green trace), whereas there was an incomplete, but substantial, inhibition of Ca^2+^ entry in the PSC (Figure 5C, red trace), in agreement with our previous findings.^11^

### Pharmacology of ATP-elicited Ca^2+^ Signaling in PMs

**Figure 6.**
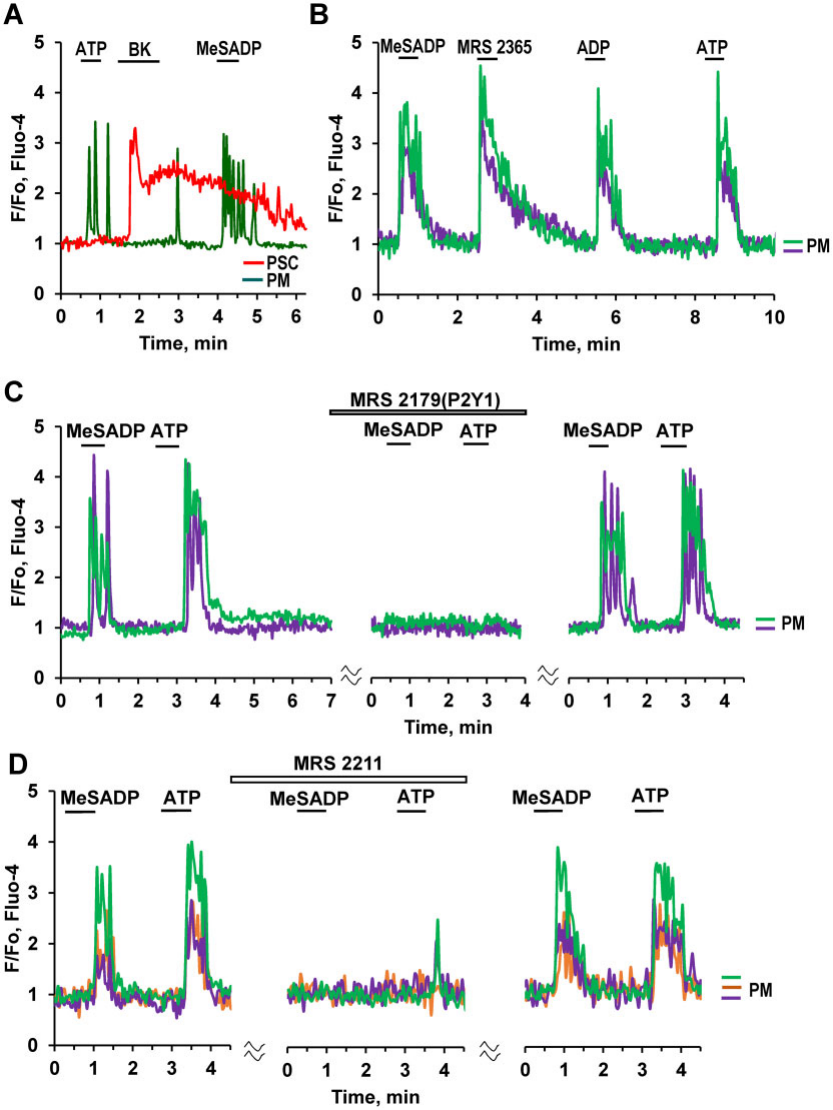
Pharmacology of ATP-elicited Ca^2+^ Signals in PMs. **(A).** Both ATP (10 μM) and MeSADP (0.1 μM) evoke Ca^2+^ signals in a PM (*n* = 64), but not in a BK-sensitive PSC. MeSADP is a potent purinergic agonist displaying selectivity for P2Y1, P2Y12, and P2Y13 (pEC50 = 8.29 and 9.05 for P2Y1 and P2Y12, EC50 = 19 nM for P2Y13). **(B).** Ca^2+^ signals in two PMs induced by subsequent addition of purinergic agonists: MeSADP (*n* = 64), the selective P2Y1 agonist MRS 2365 (0.2 μM, 10 out of 15 cells), ADP (20 μM, *n* = 16), and ATP (10 μM)**. (C).** MRS 2179, a selective antagonist of P2Y1 receptors, reversibly blocks Ca^2+^ transients evoked by activation of purinergic receptors by 0.3 μM MeSADP (8 out of 10 cells) and 10 μM ATP. **(D).** MRS 2211(10 μM), a competitive antagonist of P2Y13 receptors reversibly blocks Ca^2+^ signals evoked by both ATP (10 μM, 13 out of 14 cells) and MeSADP (0.3 μM, 11 out of 12 cells) in ΠΜσ.

The purinergic agonist MeSADP, which mainly acts on P2Y1, P2Y12, and P2Y13 receptors,^27^ elicited Ca^2+^ signals in PMs (Figure 6A–D). MRS 2179, a selective antagonist of P2Y1 receptors, completely blocked Ca^2+^ transients in PMs induced by either ATP or MeSADP (Figure 6C). However, MRS 2211, a competitive antagonist of P2Y13 receptors also blocked both ATP and MeSADP responses in PMs (Figure 6D). A selective P2Y1 agonist, MRS 2365, as well as ADP, induced Ca^2+^ signals in PMs (Figure 6B), whereas suramin, a potent blocker of P2Y purinergic receptors, blocked Ca^2+^ signals in response to application of 10 μM ATP in PMs (*n* = 3). We suggest that this may be due to co-operation between P2Y1 and P2Y13 in PMs, as previously reported for mesenchymal stromal cells in adipose tissue.^27^

### BK-elicited Ca^2+^ Signals in PMs

**Figure 7.**
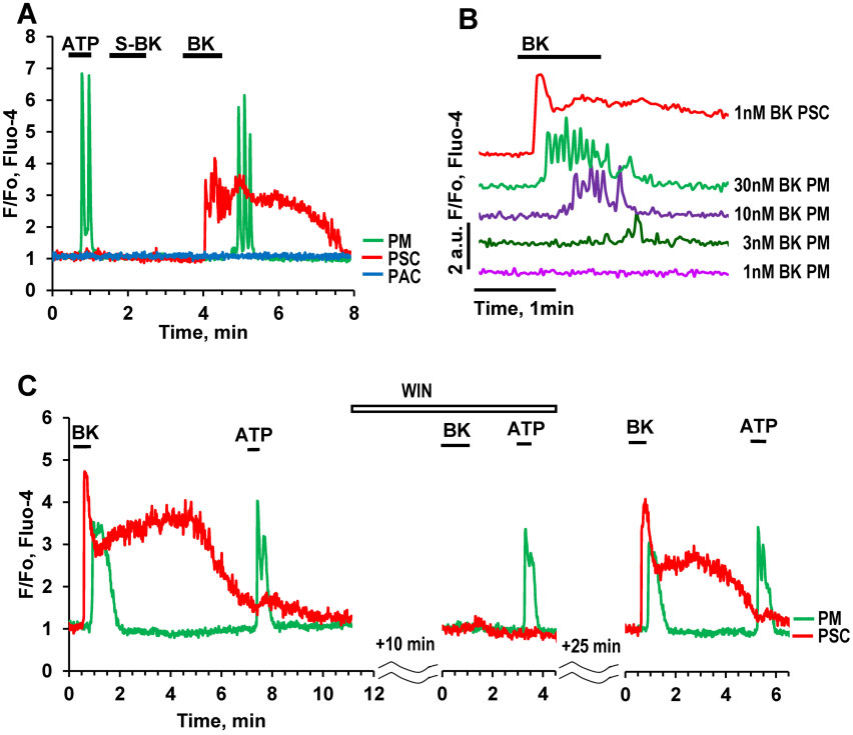
BK Concentration–Response Relationship in PMs. **(A).** Representative [Ca^2+^]_i_ trace shows that 20 nM BK can evoke a Ca^2+^ signal in a PM (but delayed compared to the Ca^2+^ signal simultaneously recorded in a neighboring PSC) (81 out of 207 tested cells) while the B_1_ type receptor agonist S-BK has no effect. **(B).** Representative traces from the same cell shows concentration-dependence of BK-induced Ca^2+^ signals in PM. For comparison, the upper trace (red) shows a BK (1 nM)-induced Ca^2+^ signal in a PSC. **(C).** The bradykinin B_2_ type receptor antagonist WIN 64338 reversibly blocks Ca^2+^ signals evoked by BK (20 nM) in both a PM (*n* = 8) and a PSC, with no effect on the ATP responses in the PM.

We have previously reported that a low concentration of BK (1 nM), which typically elicits clear Ca^2+^ signals in PSCs did not induce any changes in the cytosolic Ca^2+^ concentration ([Ca^2+^]_i_) in X-cells (PMs).^11^ However, in the present study, we found that in about 40% of PMs tested, there was a Ca^2+^ signal in response to higher concentrations of BK (Figure 7A, B). Figure 7B shows typical Ca^2+^ signals in PMs elicited by BK at concentrations from 3 nM to 30 nM, but not at 1 nM BK. The BK-induced Ca^2+^ signals in PMs were delayed as compared to those in PSCs (Figure 7A, C). The delay depended on the BK concentration and became progressively shorter at higher concentrations (Figure 7B). The B2 receptor antagonist WIN 64338 reversibly inhibited the responses to BK (Figure 7C) in both PMs and PSCs, suggesting that, similar to our previous finding for PCSs,^11^ PMs possess the type 2 BK (B2) receptor. Atropine (Figure 3C) had no effect on the BK-induced responses in PMs.

## Discussion

In the quest to understand the mechanisms governing the function of an organ, it is natural initially to focus on the dominant cell type, particularly if it is clear that it is the executor of the organ’s primary physiological task. In the exocrine pancreas, it has been clear for a long time that the quantitatively dominating acinar cells synthesize the whole range of enzymes required for the digestion of food entering the gut and that these cells secrete these enzymes in a manner precisely controlled by local repetitive cytosolic Ca^2+^ spikes.^1^ It is also clear that AP is initiated in the acinar cells by excessive global and sustained elevations of [Ca^2+^]_i_.^6^ However, in recent years it has become clear that there are other cell types in the peri-acinar environment that can be regarded as “background actors” and there is increasing interest in understanding their function and how they may interact with the principal cells.^28^ Given that cytosolic Ca^2+^ signal generation is one of the most fundamental mechanisms regulating physiological processes^29–31^ and that such signals can now be readily recorded in the quasi-intact pancreas,^10^^,^^11^ Ca^2+^ measurements in the peri-acinar cells could be an important tool for obtaining information about the signaling functions of these accessory and less common cell types.

Having recently characterized the Ca^2+^ signaling properties of the pancreatic stellate cells (PSCs) in pancreatic lobules and assessed their potential influence on the acinar cells,^10^^,^^11^ we have now turned our attention to the immune cells in the exocrine pancreatic tissue. We show that these cells exist in low density in the normal pancreas and confirm that their number increases markedly in the first few days following induction of AP by a combination of ethanol and fatty acids (Figure 2).

Our data for the first time demonstrate functional responses of immune cells in their natural environment, namely in live pancreatic tissue. Ca^2+^ measurements in *ex vivo* lobules have been complemented by immunocytochemistry to test the identity of the monitored cells.^10^^,^^11^ This technique has allowed us to study both physiological and pathological responses related to AP. Live immunocytochemistry is ideal for primary antibodies labeled with a fluorescent marker, in our case Alexa 647, but could also be carried out with the use of secondary antibodies as previously reported.^10^^,^^11^ The positive staining with antibodies against F4/80 and CD11b (Figures 1 and 2) indicates that these immune cells are most likely PMs,^32^ which have previously been reported to increase in numbers in chronic pancreatitis in mice.^20^

Macrophages are known to express a variety of purinergic P2Y receptors^33^ and here we show that both ADP and ATP activate P2Y receptors blocked by suramin and specific inhibitors of either P2Y1 or P2Y13 receptors (Figure 6). The effects of ATP and MeSADP (agonist of P2Y1, P2Y12, and P2Y13 receptors) were abolished by either MRS2179, a selective antagonist of P2Y1 receptors, or MRS 2211, a competitive antagonist of P2Y13.^27^ Similar synergistic responses have previously been described for ADP in mesenchymal stromal cells,^27^ which required both P2Y1 and P2Y13 receptors, while specific inhibition of either of them abolished the responses to ADP.

It has been known for a long time that macrophages can generate cytosolic Ca^2+^ signals via activation of IP_3_ receptors^34^ and here we show that the ATP-induced Ca^2+^ signals in PMs are primarily due to intracellular Ca^2+^ release (Figure 3) and can be very markedly reduced by the phospholipase C inhibitor U73122 or the inhibitor of store-operated Ca^2+^ entry 2-APB (Figure 4), which can also inhibit IP_3_-elicited Ca^2+^ release.^35^

It is well established that macrophages produce and secrete a wide range of inflammatory cytokines and Ca^2+^ signals in these cells have been linked to both production and secretion of these inflammatory mediators.^33^^,^^36–38^ Although we currently have no data about the consequence(s) of Ca^2+^ signal generation specifically in the macrophages in the intact pancreas, it is likely—based on what is generally known about macrophage function,^33^^,^^36–38^ that the Ca^2+^ signals described in our study could play an import role in the generation of the cytokine storm that is such an important feature of severe AP.^39^

It has very recently been noted that there are interesting similarities between the multi-organ failures and patterns of elevated cytokines observed in severe cases of COVID-19 and AP.^39^ Furthermore, it has been suggested that in addition to the cytokine storm, there is also a BK storm involved in severe cases of COVID-19.^40^ This may also be the case in severe cases of AP. Plasma levels of BK are elevated in AP,^41^ and this causes Ca^2+^ signal-mediated NO formation in the PSCs.^10–12^ There is evidence indicating that the NO formation amplifies acinar necrosis.^11^^,^^12^ In macrophages, intracellular Ca^2+^ can also activate NO production.^36^ It is therefore possible that ATP-elicited Ca^2+^ signals in the PMs, via NO formation, could participate in the vicious circle previously described in relation to the interaction between acinar and stellate cells.^11^ The cytokine storm is likely to be amplified by the BK storm, since we have shown in this study that the PMs are not only activated by ATP, but also by BK.

Initial damage of the acinar cells, elicited by combinations of fatty acids and ethanol, by bile acids or by asparaginase^6^^,^^7^^,^^11^ would release ATP into the acinar environment and elicit Ca^2+^ signals in the pancreatic macrophages. Via NO formation (and possibly also other mechanisms), further damage of the pancreatic acinar cells would occur with additional release of ATP establishing a vicious circle. An initial element of inflammation would induce entry of more PMs into the pancreatic tissue, amplifying the overall impact of Ca^2+^ signal generation in these cells and further worsening the severity of AP.

Clearly, we are still at an early stage of understanding the initiation of the inflammatory response that in severe cases of AP results in destruction of the pancreas and other organs, but we can now begin to appreciate the importance of the role that cells in the peri-acinar environment, such as stellate cells and PMs, could play in this process.

## Acknowledgments

O.G., J.V.G., O.H.P., and O.V.G. designed the study; O.G., J.V.G., and O.V.G. conducted and analyzed the experiments; O.G., J.V.G., O.H.P., and O.V.G. wrote the manuscript.

## Funding

The work was supported by grants from the Medical Research Council (UK) (MR/J002771/1 and G19/22/2 to O.H.P.) and Children with Cancer UK grants (2014/167 and 2019/288 to O.V.G. and J.V.G.).

## Conflict of interest statement

None declared.

## References

[1] Petersen OH Tepikin A. Polarized calcium signaling in exocrine gland cells. Annu Rev Physiol 2008;70:273–299. 17850212 10.1146/annurev.physiol.70.113006.100618

[2] Gerasimenko JV Gryshchenko O Ferdek PE , et al Ca^2+^ release-activated Ca^2+^ channel blockade as a potential tool in anti-pancreatitis therapy. Proc Natl Acad Sci U S A 2013;110:13186–13191. 23878235 10.1073/pnas.1300910110 PMC3740877

[3] Gerasimenko JV Charlesworth RM Sherwood MW , et al Both RyRs and TPCs are required for NAADP-induced intracellular Ca^2+^ release. Cell Calcium 2015;58(3):237–245. 26100948 10.1016/j.ceca.2015.05.005 PMC4539342

[4] Pallagi P Madacsi T Varga A , et al Intracellular Ca^2+^ signalling in the pathogenesis of acute pancreatitis: recent advances and translational perspectives. Int J Mol Sci 2020;21(11):4005. 10.3390/ijms21114005 PMC7312053 32503336

[5] Murphy JA Criddle DN Sherwood M, et al Direct activation of cytosolic Ca^2+^ signaling and enzyme secretion by cholecystokinin in human pancreatic acinar cells Gastroenterology 2008;135(2):632–641. 18555802 10.1053/j.gastro.2008.05.026

[6] Gerasimenko JV Gerasimenko OV Petersen OH. The role of Ca^2+^ in the pathophysiology of pancreatitis. J Physiol 2014;592(2):269–280. 23897234 10.1113/jphysiol.2013.261784 PMC3922492

[7] Peng S Gerasimenko JV Tsugorka TM , et al Galactose protects against cell damage in mouse models of acute pancreatitis. J Clin Invest 2018;128(9):3769–3778. 29893744 10.1172/JCI94714 PMC6118583

[8] Swain SM Romac JM-J Shahid R , et al TRPV4 channel opening mediates pressure-induced pancreatitis initiated by Piezo1 activation. J Clin Invest 2020;130(5): 2527–2541. 31999644 10.1172/JCI134111 PMC7190979

[9] Ferdek PE Jakubowska MA Gerasimenko JV , et al Bile acids induce necrosis in pancreatic stellate cells dependent on calcium entry and sodium‐driven bile uptake. J Physiol 2016;594(21):6147–6164. 27406326 10.1113/JP272774 PMC5088250

[10] Gryshchenko O Gerasimenko JV Gerasimenko OV , et al Ca^2+^ signals mediated by bradykinin type 2 receptors in normal pancreatic stellate cells can be inhibited by specific Ca^2+^ channel blockade. J Physiol 2016;594(2):281–293. 26442817 10.1113/JP271468 PMC4713750

[11] Gryshchenko O Gerasimenko JV Peng S , et al Calcium signalling in the acinar environment of the exocrine pancreas: physiology and pathophysiology. J Physiol 2018;596(14):2663–2678. 29424931 10.1113/JP275395 PMC6046068

[12] Jakubowska MA Ferdek PE Gerasimenko OV , et al Nitric oxide signals are interlinked with calcium signals in normal pancreatic stellate cells upon oxidative stress and inflammation Open Biol 2016;6(8):160149. 27488376 10.1098/rsob.160149 PMC5008014

[13] Calderon B Carrero JA Ferris ST , et al The pancreas anatomy conditions the origin and properties of resident macrophages. J Exp Med 2015;212:1497–1512. 26347472 10.1084/jem.20150496 PMC4577842

[14] Russo MW Wei JT Thiny MT , et al Digestive and liver diseases statistics, 2004. Gastroenterology 2004;126:1448–1453. 15131804 10.1053/j.gastro.2004.01.025

[15] Gundra UM Girgis NM Ruckerl D , et al Alternatively activated macrophages derived from monocytes and tissue macrophages are phenotypically and functionally distinct. Blood 2014;123 (20):e110–e122. 24695852 10.1182/blood-2013-08-520619 PMC4023427

[16] Schulz C Gomez Perdiguero E Chorro L , et al A lineage of myeloid cells independent of Myb and hematopoietic stem cells. Science 2012;336(6077):86–90. 22442384 10.1126/science.1219179

[17] Weisberg SP Carpenter DJ Chait M , et al Tissue-resident memory T cells mediate immune homeostasis in the human pancreas through the PD-1/PD-L1 pathway. Cell Rep 2019;29, 3916–3932. 31851923 10.1016/j.celrep.2019.11.056 PMC6939378

[18] Rakonczay Z Hegyi P Takacs T , et al The role of NF-κB activation in the pathogenesis of acute pancreatitis. Gut 2008;57(2):259–267. 17675325 10.1136/gut.2007.124115

[19] Wen L Voronina S Javed MA al. Inhibitors of ORAI1 prevent cytosolic calcium-associated injury of human pancreatic acinar cells and acute pancreatitis in 3 mouse models. Gastroenterology 2015;149(2):481–492. 25917787 10.1053/j.gastro.2015.04.015 PMC4556985

[20] Xue J Sharma V Hsieh MH. Alternatively activated macrophages promote pancreatic fibrosis in chronic pancreatitis. Nat Commun 2015;6:71582014. 10.1038/ncomms8158 PMC4632846 25981357

[21] Nikolic T Bunk M Drexhage HA , et al Bone marrow precursors of nonobese diabetic mice develop into defective macrophage-like dendritic cells in vitro. J Immunol 2004;173(7):4342–4351. 15383563 10.4049/jimmunol.173.7.4342

[22] Rostam HM, Reynolds PM Alexander MR , et al Image based Machine Learning for identification of macrophage subsets. Sci Rep 2017,7:3521. 28615717 10.1038/s41598-017-03780-z PMC5471192

[23] Skinner BM Johnson EEP. Nuclear morphologies: their diversity and functional relevance. Chromosoma 2017;126:195–212. 27631793 10.1007/s00412-016-0614-5 PMC5371643

[24] Trescos Y Tessier E Rougeaux C , et al Micropatterned macrophage analysis reveals global cytoskeleton constraints induced by bacillus anthracis edema toxin. Infect Immun 2015, 83:3114–3125. 26015478 10.1128/IAI.00479-15 PMC4496603

[25] Myers JT Swanson JA. Calcium spikes in activated macrophages during Fcγ receptor-mediated phagocytosis. J Leukocyte Biol 2002;72:677–684. 12377936

[26] Feske S Gwacket Y Prakriya M , et al A mutation in Orai1 causes immune deficiency by abrogating CRAC channel function. Nature 2006;441:179–185. 16582901 10.1038/nature04702

[27] Kotova PD Bystrova MF Rogachevskaja OA et al . Coupling of P2Y receptors to Ca^2+^ mobilization in mesenchymal stromal cells from the human adipose tissue. Cell Calcium 2018;7:1–14. 10.1016/j.ceca.2017.11.001 29604959

[28] Kusiak AA Szopa MD Jakubowska MA , et al Signaling in the physiology and pathophysiology of pancreatic stellate cells – a brief review of recent advances. Front Physiol 2020;11:78. 32116785 10.3389/fphys.2020.00078 PMC7033654

[29] Berridge MJ. The inositol trisphosphate/calcium signaling pathway in health and disease. Physiol Rev 2016;96:1261–1296. 27512009 10.1152/physrev.00006.2016

[30] Petersen OH Verkhratsky A. Calcium and ATP control multiple vital functions. Philos Trans R Soc Lond B Biol Sci 2016;371(1700):20150418. 27377728 10.1098/rstb.2015.0418 PMC4938019

[31] Rizzuto R Pozzan T. Microdomains of intracellular Ca^2+^: molecular determinants and functional consequences. Physiol Rev 2006;86(1):369–408. 16371601 10.1152/physrev.00004.2005

[32] Galli SJ Borregaard N Wynnet TA. Phenotypic and functional plasticity of cells of innate immunity: macrophages, mast cells and neutrophils. Nat Immunol 2011;12(11):1035–1044. 22012443 10.1038/ni.2109 PMC3412172

[33] Desai BN Leitinger N. Purinergic and calcium signaling in macrophage function and plasticity. Front Immunol 2014;5:580. 25505897 10.3389/fimmu.2014.00580 PMC4245916

[34] Randriamampita C Bismuth G Trautmann A. Ca^2+^-induced Ca^2+^ release amplifies the Ca^2+^ response elicited by inositol trisphosphate in macrophages. Cell Regul 1991;2:513–522, 1782213 10.1091/mbc.2.7.513 PMC361841

[35] Bootman MD Collins TJ Mackenzie L , et al 2‐Aminoethoxydiphenyl borate (2‐APB) is a reliable blocker of store‐operated Ca^2+^ entry but an inconsistent inhibitor of InsP_3_‐induced Ca^2+^ release. FASEB J 2002;16(10):1145–1150. 12153982 10.1096/fj.02-0037rev

[36] Feske S Wulff H Skolnik EY , et al Ion channels in innate and adaptive immunity. Annu Rev Immunol 2015;33:291–353 25861976 10.1146/annurev-immunol-032414-112212 PMC4822408

[37] Murray RZ Stow JL. Cytokine secretion in macrophages: SNAREs, Rabs, and membrane trafficking. Front Immunol 2014;5:538. 25386181 10.3389/fimmu.2014.00538 PMC4209870

[38] Kelly EK Wang L Ivashkiv LB. Calcium-activated pathways and oxidative burst mediate zymosan-induced signalling and IL-10 production in human macrophages. J Immunol 2010;184:5545–5552. 20400701 10.4049/jimmunol.0901293 PMC3016855

[39] Hegyi P Szakacs Z Sahin-Toth M. Lipotoxicity and cytokine storm in severe acute pancreatitis and COVID-19. Gastroenterology 2020;824–827. 32682765 10.1053/j.gastro.2020.07.014 PMC7366088

[40] Garvin MR Alvarez C Miller JI , et al A mechanistic model and therapeutic interventions for COVID-19 involving a RAS-mediated bradykinin storm. eLife 2020;9:e59177. 32633718 10.7554/eLife.59177 PMC7410499

[41] Hirata M Hayashi I Yoshimura K , et al Blockade of bradykinin B2 receptor suppresses acute pancreatitis induced by obstruction of the pancreaticobiliary duct in rats. Br J Pharmacol 2002;135:29–36. 11786477 10.1038/sj.bjp.0704462 PMC1573123

